# Deepening the participation of neurodivergent youth in qualitative mental health research: Co‐development of a general approach and the evaluation of its implementation in a study on emotion

**DOI:** 10.1002/jcv2.12287

**Published:** 2024-12-07

**Authors:** Myrofora Kakoulidou, Georgia Pavlopoulou, Susie Chandler, Steve Lukito, Maciej Matejko, Isabel Jackson, Beta Balwani, Tiegan Boyens, Dorian Poulton, Luke Harvey‐Nguyen, Zoe Glen, Archie Wilson, Elisa Ly, Elizabeth Macauley, Sylvan Baker, Georgina Bullen, Edmund J. S. Sonuga‐Barke, RE‐STAR team

**Affiliations:** ^1^ Department of Child & Adolescent Psychiatry Institute of Psychiatry, Psychology & Neuroscience King's College London London UK; ^2^ Group for Research in Relationships and NeuroDiversity (GRRAND) Department of Clinical, Educational & Health Psychology, Division of Psychology & Language Sciences Faculty of Brain Sciences University College London London UK; ^3^ Anna Freud London UK; ^4^ Royal Central School of Speech & Drama London UK; ^5^ University of Greenwich London UK

**Keywords:** ADHD, autism, co‐production, neurodivergence, participatory research, patient public involvement, qualitative

## Abstract

**Background:**

There is a growing expectation that neurodivergent young people, such as those with diagnoses of attention‐deficit/hyperactivity disorder (ADHD) and/or autism, should play a central role in shaping research on neurodevelopmental conditions. However, currently, their involvement is typically limited to arms‐length advice. To address this, the *Regulating Emotions‐Strengthening Adolescent Resilience* (RE‐STAR) programme has co‐developed a framework for deepening the involvement of neurodivergent participants in translational research. Here we apply this to build, implement and evaluate a new approach to participatory qualitative research.

**Methods:**

Development – Building on the track record of successful collaboration between RE‐STAR academic researchers (ARs) and its Youth Researcher Panel (Y‐RP), a cycle of meetings was convened to co‐develop a collaborative protocol for the participatory approach. Implementation – ARs and Y‐RPers applied the general protocol to study a specific topic. This involved co‐designing and co‐delivering an interview schedule to study the emotional experiences of 12 adolescents with diagnoses of ADHD and/or autism and then co‐analysing the interviews. Evaluation – ARs, Y‐RPers and interviewees shared their reflections on the participatory approach and its implementation, during interviews (*N* = 36) and short open‐ended surveys (*N* = 22).

**Results:**

Development ‐ The protocol for the participatory approach gave detailed advice on how to engage Y‐RP members (or equivalent) in the co‐design, co‐delivery and co‐analysis of interviews. Implementation ‐ The approach was successfully implemented by ARs and Y‐RPers working together to co‐design an interview to study the emotional lives of adolescents with diagnoses of ADHD and/or autism, co‐deliver it and then co‐analyse the interview scripts. Evaluation ‐ The implementation experience of the Y‐RP, ARs and interviewees was characterised by common themes relating to (a) adapting research methods and making practical adjustments; (b) taking on new roles, adding value to research and (c) valuing neurodivergent characteristics.

**Conclusions:**

This new RE‐STAR protocol proved feasible to implement in a way that was generally perceived, from multiple perspectives, to add depth and authenticity to research into the experiences of neurodivergent young people.

**Key points**

**What's known?**

- The participation of neurodivergent people is encouraged in research on attention‐deficit/hyperactivity disorder (ADHD) and autism but often restricted to arms‐length advice from Patient and Public Involvement (PPI) committees outside the core research team. The *Regulating Emotions ‐ Strengthening Adolescent Resilience* programme has developed a deeper participatory framework that places young people with diagnoses of autism and ADHD at the heart of the full research cycle.

**What's new?**

- Here we apply this general framework to co‐develop and successfully co‐deliver a protocol of participatory qualitative research involving the co‐design of a new interview schedule, its co‐delivery and the co‐analysis of scripts.

**What's relevant?**

- Including young people with diagnoses of ADHD and autism in research has the potential to revolutionise our understanding of their mental health needs and, therefore, advance clinical practice.

## INTRODUCTION

Valued by patient communities and researchers and mandated by funders, Patient and Public Involvement (PPI) is now considered standard practice in mental health research (Denegri et al., 2015; Involve, 2019; Oliver et al., 2004; Richmond et al., 2023). In line with this, people diagnosed with attention‐deficit/hyperactivity disorder (ADHD) and/or autism are routinely consulted on the conduct of research into their conditions (Fletcher‐Watson et al., 2021; Pellicano et al., 2014). Involving neurodivergent people in research can bridge the research‐to‐practice “relevance gap”, democratise the research process (Beresford & Croft, 2012) and encourage ethical practice, creating solutions aligned with these communities' priorities and needs (Cage et al., 2024; Pellicano & den Houting, 2022). However, PPI is typically constituted *outside* the core research team (den Houting et al., 2021) with input restricted to more marginal research‐related tasks (e.g., research documentation design, participant engagement approaches, intervention acceptability).

In response to these concerns, there are growing calls for inclusive research frameworks (Fletcher‐Watson et al., 2019, 2021) that will deepen and broaden the participation of neurodivergent people in research (Nicolaidis et al., 2019)—making them a core part of the research processes where they can improve research across the full cycle of investigation (Sonuga‐Barke, 2023). Key principles underpinning this sort of ‘co‐production’ include (a) power‐sharing; (b) inclusion of all perspectives and skills; (c) reciprocity; and (d) the building and maintaining of relationships (Hickey et al., 2018). Studies of neurodivergence using co‐production have started to emerge (e.g., Flobak et al., 2021; Keating, 2021; Nicolaidis et al., 2019; Pavlopoulou, 2021) following the research tradition in health research (Brett et al., 2014; Cornwall & Jewkes, 1995; Epstein, 1996). Crompton et al. (2022) collaborated with autistic people to co‐design an interview schedule to understand autistic people's experiences of peer support. Pellicano et al. (2022) collaborated with autistic scholars and advocates to deliver and analyse interviews of late‐diagnosed autistic adults. Flobak et al. (2021) reported a collaboration between academics and ADHD patients in the co‐design of video vignettes for an online mental health intervention.

Systematic evaluations of the benefits and impact of co‐production approaches are required, but often absent (Bennett et al., 2022). Conventional PPI approaches, although enthusiastically implemented by academic researchers (ARs), are often perceived as tokenistic by members of the patient community (Paul & Holt, 2017). With regard to deeper co‐production, Pellicano et al. (2022) found that autistic participants were very positive about being interviewed by fellow autistic researchers, however, the views of the researchers were not sought in this case. Davies et al. (2024), asked autistic researchers and participants about their views on co‐designing a peer support programme; however, the focus was on improving the programme rather than the co‐production process. Thus, it remains unclear how participation impacts neurodivergent people and what are the most effective ways to involve them in research.

This paper describes an interdisciplinary research programme, *Regulating Emotions—Strengthening Adolescent Resilience* (RE‐STAR), pioneering a deeper participatory framework with neurodivergent young people at the heart of the scientific process and core members of the team (Sonuga‐Barke et al., 2024). Its goal is to develop new school‐based interventions to reduce the risk of depression in neurodivergent adolescents. Informed by the concept of co‐intentionality (Freire, 1970), this framework aims for an even‐handed collaboration between neurodivergent young people and ARs, who despite having different perspectives, experiences and expertise, work together towards a common goal. Pivotal to this process is the Youth Researcher Panel (Y‐RP; individual members referred to here as “Y‐RPers”): Originally 10 young adults (aged 18—25 years) with diagnoses of ADHD and/or autism formed RE‐STAR's Y‐RP. Over time, with appropriate training and support, the Y‐RPers have gradually taken on a fuller co‐researcher role and become increasingly integrated within the core research team. This gives them the potential to influence RE‐STAR across the entire scientific cycle (see Sonuga‐Barke et al., 2024 for a detailed description of the framework), including bringing new perspectives to shape theory development and the specification of hypotheses, contributing to new methods and co‐producing research materials, undertaking data collection and bringing insights to aid the interpretation and dissemination of findings.

The objectives of the current paper are.

1. To describe how Y‐RPers and ARs worked together using the RE‐STAR participatory framework to develop a general protocol for co‐production in qualitative research studies of ADHD and autism.
2. To describe the implementation of this general protocol by RE‐STAR ARs and Y‐RPers to study a specific topic: The links between neurodivergence, emotion and mental health in adolescents with ADHD or autism diagnoses.
3. To evaluate the acceptability and value of the general protocol from the perspectives of the Y‐RPers, neurodivergent interviewees and ARs involved in its specific implementation in the study.

## METHODS

### Ethics

The study received ethical approval from King's College London's Research Ethics Committee (ref. HR/DP‐21/22‐29361) and was conducted by the principles of the Declaration of Helsinki. Informed parental consent/young person assent was secured. Both Y‐RP and academic co‐interviewers gave written consent.

### Participants

At the time of the study, the Y‐RP group consisted of 10 young people with diagnoses of ADHD (*n* = 2), autism (*n* = 5) or both ADHD and autism (*n* = 3) aged between 18 and 25 years. All Y‐RPers and five ARs (*n* = 3 multiply neurodivergent with a range of co‐occurring diagnoses such as ADHD, dyslexia and dyspraxia) were involved in general protocol development. Ten Y‐RPers and five ARs worked together to co‐design the interview. Six Y‐RPers and two ARs were co‐interviewers. Four Y‐RPers and four ARs were involved in the co‐analysis phase.

The interviewees in the implementation phase were 12 adolescents attending mainstream schools, aged 11–15 years, recruited through RE‐STAR's partner charities and via newsletters and advertisements on social media. Six had an autism diagnosis, three had an ADHD diagnosis and three had both ADHD and autism diagnoses. All interviewees had sufficient use/understanding of spoken English. Interviews took place over 5 months.

### Procedure

#### Development

The co‐produced general protocol involved 21 scheduled group meetings and many one‐to‐one encounters using Padlet, PowerPoint, Word docs and email activities undertaken over 30 months. Each group meeting typically lasted for 90 min. Reflexive diaries completed during meetings provided the basis for subsequent meetings to ensure an iterative approach to the development of *the protocol*. The goals of these meetings were to; (a) agree on the aims and objectives of the research topic and discuss pre‐study considerations; (b) agree on the scope of the general protocol; (c) establish ground rules for Y‐RP participation to create equitable and effective partnerships by identifying communication, training and participation needs for both Y‐RPers and ARs; and (d) think through how the Y‐RPers’ role should be defined and facilitated. The following approach was agreed for Y‐RP involvement in the co‐design, co‐interviewing and co‐analysis stages. *More details of the specific elements included in the general protocol are included in Part 1 of the results section.*

***Interview co‐design element***: During early Y‐RP gatherings, the importance of using interactive visual arts techniques and thinking boards (e.g., Padlet) to gather Y‐RPer insights and concepts was highlighted. Arts‐based methods included poetry, imaginative writing, photography, sketching and drawing. This was important as it enabled neurodivergent people to collectively explore the various viewpoints, nuances and personal experiences. This allowed generated themes from Y‐RPer responses, not necessarily considered a priori, to be included in an interview schedule. Additionally, it gave participants with different processing styles the opportunity to choose for themselves in advance, what and how they wanted to communicate.

***Co‐interviewing and co‐analysis elements:*** Motivated by the Y‐RPers’ expressed interest in being involved in data collection and analysis, it was agreed that the general protocol would be extended to include guidance on these elements. To this end, several meetings and activities were set up to discuss how these could be best achieved in general and more specifically issues around research ethics and safeguarding for Y‐RPers and interviewees. The specifics of the interviewer and analysis roles for ARs and Y‐RPers were agreed in a series of group and one‐to‐one meetings. Disagreements were resolved through discussions, with precedence being given to the views of the Y‐RPers. Training resources and practice needs were identified.

Y‐RPers were paid for their involvement in the protocol co‐development in line with NIHR guidelines (2024).

#### Implementation

The general protocol was applied to the specific topic of emotions over 6 months. The Y‐RPers and ARs worked together to co‐design and deploy, and then co‐analyse the transcribed interview scripts generated from an interview schedule exploring the emotional lives of adolescents with diagnoses of ADHD and/or autism. A series of meetings were also organised to introduce the Y‐RPers to interviewing and qualitative research techniques. After this, a working team of four Y‐RPers took on the role of co‐analysts alongside four ARs. During further small group meetings, one‐to‐one meetings and email conversations, the full team agreed on the co‐analysis methods and timeline, and the specific role each person would play.

Each adolescent interviewee took part in two sessions, an orientation session in which the researchers explained the study aims and interview procedures and checked for reasonable adjustments, and the actual interview, lasting approximately 30 and 90 min, respectively.

#### Evaluation

Each step of the research process involved in the implementation (interview co‐design, co‐interviewing and co‐analysis) was examined using semi‐structured feedback interviews and a brief survey with open‐ended questions to gauge Y‐RPers’, ARs' and interviewees' experiences. These feedback interviews were delivered by two RE‐STAR ARs (SC and GB) who had not been involved in the co‐interviewing. A range of common topics was covered with Y‐RPers and ARs (e.g., support and training needs, experience of the process, strengths and weaknesses of the approach). Interviewee questioning focussed on their experiences of the interviews and the value of being interviewed by a neurodivergent researcher. Thirty‐six post‐implementation interviews were conducted and transcribed. The Y‐RPers and ARs also completed a short survey about their training and co‐analysis experiences and perceived strengths and challenges to this model of co‐production. The survey questions required a free‐text format response (see supporting information for the survey questions). Y‐RPers and ARs also provided written reflections on their co‐analysis experience.

All outputs were coded and analysed using inductive‐thematic approaches (Braun & Clarke, 2019) and drawing upon a “critical realist” perspective (Terry et al., 2017). Two authors (one neurodivergent and one neurotypical) read and independently coded the text data. Together, they generated codes at a semantic level and clustered these into themes that tapped into shared meanings. The codes and themes were then shared with three Y‐RPers and the team worked together on the final organisation.

#### Positionality and reflexivity

During the co‐analysis of the main interviews, Y‐RPers and ARs discussed their subjective perspectives on the research topic in weekly/monthly research meetings. Before coding, the team dedicated time to answering prompts related to their relationship with research methods and experiences of neurodivergence and mental health. This encouraged recognition of the “positionality“ each brought to the research and their familiarity with the research topic on both a scientific and personal level. This approach may be superior to the use of “unknowledgeable” coders, who may lack the richness that insider researchers often bring to the coding process (Morse et al., 2002).

Throughout the analysis, we explored our diverse neurodivergent identities, the multiple realities (family and school experiences) and the different epistemologies we brought to the multi‐disciplinary team and how they influenced our notions about ADHD and autism and our research approaches. We discussed different ways of coding and analysis following guidance by Campbell et al. (2021). Y‐RPers led the dissemination plans and shared their experiences through events and talks (Sonuga‐Barke et al., 2024).

## RESULTS

The results are presented in three parts. Part 1 describes in detail *the general protocol* co‐developed by Y‐RPers and ARs. Part 2 reports the implementation of *the protocol* in a co‐designed qualitative study of the emotional lives of adolescents with diagnoses of ADHD and/or autism. Part 3 presents the findings from the analysis of the post‐implementation qualitative interviews and surveys.

1. The general protocol

The general protocol is a guide, built by the Y‐RPers and ARs, to support co‐produced neurodivergent youth‐led participatory qualitative research. It includes guidance on three elements—interview co‐design, co‐interviewing and co‐analysis over six phases (see Table 1). *Phase one* involves ‐ agreeing on ground rules for collaboration; exploring the emotional experiences of neurodivergent Y‐RP co‐researchers related to the topic under investigation; setting the scope and defining terms and concepts. *Phase two* includes the interview co‐development stages based on themes derived from neurodivergent Y‐RP's experiences and the existing literature on the topic of interest. It also includes the development/selection of alternative approaches for eliciting information from neurodivergent people (Pavlopoulou, 2021). *Phase three* involves guidance for the preparation for co‐interviewing‐training, role exploration/agreement and script finalisation. *Phase four* relates to the actual co‐interviewing process. It recommends two sessions: an orientation and a main interview session recognising the need for ample preparation and debriefing. *Phase five* describes the preparation for co‐analysis through familiarisation with the transcripts and coding processes. This took place during meetings with Y‐RP‐ARs pairings and the whole group. *Phase six* provides guidance on interview co‐analyses and the generation of codes and themes with a selection of quotes.

2. Implementation

**TABLE 1:** Co‐developed protocol for participatory qualitative research across all phases.

Phase	Step
1 General preparation	● Establish ground rules, agree involvement expectations. ● Y‐RP share preferred ways of communication, define how an equitable partnership may look like and discuss preferred ways of reviewing partnership and feedback. ● Co‐produce a *Duty of Care Protocol* (available%20here). ● Y‐RP share experiences related to topic through discussions and multimodal activities (e.g., Padlet) with ARs. ● Co‐establish scope & focus of study; agree key research terms/definitions using creative activities in synchronous and asynchronous activities. ● Co‐design information letters, consent/assent forms and co‐interviewing guidelines.
2 Interview co‐development	● ARs identify themes in Y‐RP experience descriptions to structure interview schedule. ● Y‐RP offer feedback on themes and AR make adjustments. ● Y‐RP and ARs translate themes into interview topics and questions. ● Co‐design participant‐led tasks to facilitate discussion of topics (creative task and video vignettes).
3 Co‐interviewing preparation	● ARs train Y‐RPers in interviewing methods, ethics and safeguarding. ● Y‐RPers practice interview schedule in role‐play activities. They also practice how to use interview prompts and active listening. ● Y‐RPers & ARs form co‐interviewing pairs, practice delivery, agree roles and adjust. ● ARs and YRPers together finalise ethics documents/interview schedule/guidelines. ● Y‐RPs complete GDPR training (King's College London). ● Open call for co‐interviewers—Six Y‐RP decide to take on the co‐interviewer role. ● Six Y‐RPs and two ARs pilot the interview schedule.
4 Co‐interviewing	● One Y‐RP interviewer and one academic interviewer co‐deliver two separate sessions with a participant. ● Orientation session: Explain interview procedures, discuss reasonable adjustments and set creative ‘homework’ (e.g., drawing, collage and lego modelling) to provide a focus for discussion in .Session 2 ● Main interview session: Together deliver the co‐developed interview schedule. ● Before sessions, Y‐RPers and ARs prepare together, and after sessions reflect on their interview experience.
5 Co‐analysis preparation & data familiarisation	● Y‐RPers are trained in relevant qualitative methods after a period of practising coding exercises in full team meetings. ● Y‐RPers choose to get involved in different analysis tasks (e.g., transcribing interviews, coding transcripts) supported by AR. Y‐RPers and ARs code one or two transcripts individually. ● During meetings, Y‐RPers and ARs discuss codes and practice reflexivity/discuss how the self they bring in research may affect coding (e.g., being neurodivergent or neurotypical).
6 Codes & themes co‐generation	● Distribute transcripts to Y‐RPer and AR pairs who code in parallel. ● Y‐RPer and ARs pairs meet online to discuss codes and reflect on their coding. ● Following discussions with Y‐RPers, ARs update the table of quotes for each transcript (e.g., renew codes, cluster codes). ● Representative quotes identified for each transcript. ● Full team meets to discuss the updated tables and generate initial themes (in progress).

Abbreviations: AR, Academic researcher; Y‐RP, Youth Researcher Panel (also called “Y‐RPers”).

*Co‐design of interviews with Y‐RPers by applying the general RE‐STAR participatory framework:* The specific research focus on emotional experiences was jointly agreed by Y‐RPers and ARs following the use of participatory arts‐based methods (poetry, photography, drawing and creative writing) and multimodal forms of communication (e.g., Padlet) to scope the study (see supporting information). The themes generated from these discussions informed the *co‐design* of an age‐appropriate interactive, experience‐sensitive interview schedule. The interview co‐design phase took place over approximately 8 weeks (see supporting information for the topic of guide/question route). Furthermore, Y‐RPers guided ARs to co‐design and pilot creative tasks from a neurodivergent perspective such as drawing, poetry and vignettes about neurodivergent‐relevant emotional situations to facilitate discussions with participants about emotional responses.

*Y‐RPer‐AR co‐delivery of interviews with adolescent participants:* Y‐RPer‐AR preparation for *co‐interviewing* involved three meetings with the whole group during which the interviews were practiced and different scenarios around ethical dilemmas and self‐care during and after the interviews, were role played. Small‐group activities and separate meetings between Y‐RPer‐AR pairs were ran to discuss how best to co‐deliver the interview schedule and support each other with mock interviews.

*Y‐RPer‐AR co‐analysis of interview transcripts:* The Y‐RPers and ARs implemented the interview *co‐analysis* process set out in Table 1. The ARs (GP and MK) generated preliminary themes based on collective coding with the Y‐RPers. The Y‐RPers attended four training meetings in reflexive thematic analysis—judged to be the most appropriate form of analysis given the goals of the study—using the framework by Braun and Clarke (2019).

3. Evaluation

This section presents the evaluation of the specific application of the general protocol in terms of themes generated across; (a) the qualitative surveys with Y‐RPers and ARs about their experiences of co‐designing the interview schedule and co‐analysing the interviews with adolescent interviewees and (b) the qualitative interviews with Y‐RPs, ARs and interviewees about their experiences of interview co‐delivery. Analysis of the evaluation themes was led by MK and GP. We generated four themes with some themes specific to the perspectives of particular participants (e.g., Y‐RPers, ARs or interviewees; see Table 2).

**TABLE 2:** Four over‐arching themes and sub‐themes across all phases.

Themes	Sub‐themes
**1: Pre‐conditions for genuine collaboration between academic and Y‐RP researchers**	Trust, mutuality & attunement Autonomy & flexibility
**2: Working together in neurodiversity‐inclusive ways**	Engaging through experience‐sensitive activities Learning from others A sense of comfort & validation
**3: Taking on new challenges & Managing struggles**	Shifting power dynamics Embracing diverse ways of thinking & working Managing duty of care, planning time & expectations
**4: Improving translational research**	Developing new research hypotheses Collecting nuanced & novel data

### Theme 1: Pre‐conditions for genuine collaboration between academic and Youth Researcher Panel researchers

Y‐RPers and ARs identified pre‐conditions needed to ensure the involvement of neurodivergent young people in research. There were two sub‐themes (a) trust, mutuality and attunement and (b) autonomy and flexibility. In the first sub‐theme, Y‐RPers described the interview co‐design as a “collaborative and validating experience”. Both Y‐RPers and ARs highlighted the need for open and safe spaces, where all voices are heard and respected.

*It quickly became clear nothing was off the table with me being allowed to discuss anything even if it couldn’t lead anywhere. There was so much willingness and readiness to listen to us and take on board what we said. It was good we were given some foundations to build up from as it gave guidance…so didn’t feel completely lost in this process and had somewhere to start…It felt a very safe space were everyone’s experience and how they shared that was valid.* (Y‐RPer)

ARs said co‐interviewing with a Y‐RPer helped create a less formal and more relaxing atmosphere encouraging interviewees to feel heard and accepted.

*…It was a great help because the participant liked Minecraft and my co‐interviewer has a little bit of knowledge about Minecraft himself and it was an avenue for us to show our appreciation of the participants' interests…* (Academic researcher)

The Y‐RPers valued their involvement in the co‐design process, the balanced structure, *autonomy and flexibility* in the interview scheduling and their training. They felt this helped them feel more “integrated” into the overall research process and that seeing how their comments and feedback translated into interview items made them feel like a “proper member of the research team”. This in turn helped them to feel more confident about co‐interviewing.

*I like the fact that we got to… be involved in the construction of the actual interview schedule. I think that meant that it was familiar. It meant that we understood what the items were that we're looking for, how they were written, how to apply them.* (Y‐RPer)

The Y‐RPers valued the collaboration with ARs, the “good interaction” during co‐interviewing (“good at handing questions off to each other”) and the support they received before, during and after interviewing. They appreciated being paired with an experienced academic interviewer. They felt “reassured” when their suggestions to improve the interview experience were accepted.

*I felt if I forgot something, or didn't ask something in the schedule, they would like notice it and maybe ask it for me as well… They were very patient and they listened to my suggestions. And anytime I have any questions, they respond to them.* (Y‐RPer)

### Theme 2: Working together in neurodiversity‐inclusive ways

This theme includes the personal benefits of neurodiversity‐informed collaborations from different points of view. It includes three sub‐themes: i) engaging in experience‐sensitive activities; ii) learning from others and iii) developing a sense of comfort and validation. ARs and Y‐RPers noted the value of creating safe spaces for the Y‐RPers and offering them opportunities for *engaging in experience‐sensitive activities*—by providing visual support, allowing supportive silence and facilitating multimedia communication. They were deemed critical to creating novel interview items.

*We discussed different ideas and we ensured we made space for Y‐RPers to decide the format. I had never worked on video vignettes in interviews before … It helped interviewees to identify and talk more about topics that otherwise are hard to introduce and get them to express themselves* (Academic researcher)

All interviewees agreed they were asked questions relevant to their emotional lives and enjoyed *engaging through experience‐sensitive activities*. Having the time to prepare the pre‐interview tasks and interacting with multimedia tools (e.g., vignettes) designed by the Y‐RPers made it easier for them to talk about their emotions. It also helped them initiate their own ideas. They felt video vignettes and emojis were helpful stimuli because they were “*some kind of relatable experiences”.*

*I liked all of it to be honest. I liked the bits where you could watch videos… it gives you more of an idea of what to talk about.* (Interviewee)

By comparing their experiences with the interviewees', the Y‐RPers felt they facilitated *learning from others,* self‐reflection and self‐understanding of their personal neurodivergence.

*As people who often feel pathologized by neurotypical researchers, this felt like opening up a true dialogue. I think we all learned a lot about ourselves by trying to learn about each other.* (Y‐RPer)

Interviewees reported *feeling more comfortable* and less lonely in a neurotypical world when answering questions in the presence of a neurodivergent Y‐RP co‐interviewer who “has been in their shoes”.

*…Sometimes I feel like only one in the world only person facing this. But (it) made me feel like people … had been through this before…They’ve obviously gone on to do great stuff.* (Interviewee)

They felt reassured that Y‐RPers would “sympathise”, which helped them feel “safe” and open up more about their emotional lives.

*I think [having a Y‐RP interviewer] helps people who are being interviewed to feel more comfortable in the interview because, you know, someone there who has probably been through near‐enough the same experiences as you. And it helps you feel easier to open up about it because it helps you feel more comfortable.* (Interviewee)

### Theme 3: Taking on new challenges and managing struggles

The Y‐RPers and ARs identified facing difficulties such as: i) shifting power dynamics; ii) embracing diverse ways of thinking and working and iii) managing duty of care, planning time and expectations.

Both the Y‐RPers and ARs reported working together to make a shift to ensure more genuine collaborations—a more even power dynamic.

*The academic researchers worked hard to re‐imagine what co‐produced research in this area might look like, advocating for the Y‐RP with stakeholders, providing frequent opportunities to feedback or take part in creating material for the interview schedule, and fostering discussions on the roles of the Y‐RP, academic researchers, and the evolving power dynamic between us.* (Y‐RPer)

*It felt essential to start with a consultation model, maybe it was good for us, it felt a safe starting point. But we were clear we did not want to stay there. Making explicit calls for Y‐RPs to take action helped us to make a shift. They needed lots of structure but also lots of freedom as well in order to take action. We managed that by giving them extra time to process and decide on our calls for action.* (Academic researcher)

ARs found co‐delivering the interview schedule with a Y‐RPer a valuable learning experience. ARs expressed very positive feelings about working with them. *Embracing diverse ways of thinking and working* was a relatively new space to navigate as they had been working solo during interviews and had developed their personal style of running them in an earlier RE‐STAR phase.

*I felt more comfortable during the shared‐experience interviews…It is relaxing to interview with someone else and have support…However, these are uncharted waters, and there is a level of uncertainty. As academic researchers, typically we are not trained to interview with other people. We are expected to take on the full control when it comes to interview delivery.* (Academic researcher)

Working within a neurodiverse team was perceived as a valuable learning experience, offering space for different ways of thinking and analysis to unfold.

*It was a very insightful experience as I heard from a very rounded discussion where there were different objectives and viewpoints…It helped me…keeping both a neurotypical and neurodivergent viewpoint and process of thinking when going with these codes, adding that personal but also professional [element] to the task.* (Y‐RPer)

*Neurodivergent young people offer new, different skills, all very useful for qualitative research.* (Academic researcher)

Both Y‐RPers and ARs acknowledged that *managing duty of care, planning time and expectations* to build an equal partnership “is not an easy road” as this “requires time, mutual trust and open dialogs”.

*It is really lots and hard work. It comes from a place of love to be able to commit and do it…, honestly you gotta love this mission, you’ve got to respect and listen and try over and over again to get it right as we learn from each other‐ in a way that you are only prepared to do for things you really love, things you really value… There is also the element of care, not to re‐traumatise anyone, to give space, to give time and to always check if you got it right. It is not easy and there is no way to simply get it right. I leant how to say sorry, how to be proactive and how to check for transparency on every step.* (Academic researcher)

Creating their own pathway for deepening the involvement of young people in mental health research, “felt like stepping into a new world” and there was a level of uncertainty, including navigating formal ethical procedures in a system that “is so unfamiliar with requests of involvement beyond PPI”.

*At first it was quite nerve‐wracking coming into a research environment and engaging with researchers… was especially noticeable when working on the pre‐study considerations as…I wasn’t sure what we could adapt or change. It was a really interesting process though …it was the first chance to really delve into the research process and put our voices into action.* (Y‐RPer)

*It was quite tricky getting HR, and the ethics committee to understand the role of the co‐interviewers, e.g., that they weren’t research participants, nor were they members of the research staff. I got the impression they had not experienced this kind of co‐production, and we were navigating a whole new route.* (Academic researcher)

The Y‐RPers underlined the need for clear timelines for training and interviewing, although acknowledging that the research process can be unpredictable at times and appreciating the team's effort to communicate changes early. Additionally, all Y‐RPers found the training useful, however, they said more practice would give them more confidence. At the same time, ARs discussed practical challenges around workload and time needed to foster and develop meaningful Y‐RP involvement.

*I think it's just about clarity and I think awareness of it did take quite a time. Which I understand completely, but I think even I find that sometimes hard*… *But I think a lot of it we've been learning as we go along.* (Y‐RPer)

*Getting a structure that works for all was a challenge at times. We had to work late in the evenings, we had to use multiple means of communication and it added a considerable amount of work. That said, it made sense to practice reasonable adjustments. We were rewarded by seeing Y‐RPs gaining more confidence and offering more back to the team.* (Academic researcher)

Finally, the ARs discussed the importance of clear duty‐of‐care procedures and setting realistic expectations at an early stage. During the interview, they felt responsible for managing time and pace, ensuring that they offered enough space and support for interviewees and Y‐RPers while also keeping the structure and following the duty of care protocol.

Interview co‐analysis was regarded as challenging.

*Sometimes [co‐analysis in groups was] tricky…especially when it came to deadline making as we all had other commitments and lengths of time a task like this would take. When it was just pairs it was a lot simpler.* (Y‐RPer)

*It was stressful for researchers to create the time, the resources and to follow up. It was hard to estimate how much or little to prepare*… *[We] had to be dynamic and follow the flow of the team.* (Academic researcher)

*Balancing the need for predictability and a dynamic reflective process in groups of 10‐15 people was not possible, yet stressful at times.* (Academic researcher)

### Theme 4: Improving translational research

The Y‐RPers and ARs acknowledged research benefits including: (a) developing new research hypotheses and (b) collecting nuanced and novel data.

Both groups reported how listening to young people can help promote alternatives to a dominant neuronormative narrative and *develop new research hypotheses*. Some provided their vision for a new field of enquiry that finds ways to support the inclusion of neurodivergent perspectives in knowledge production and which questions the theoretical and conventional assumptions that produce the idea of the neurotypical.

*I think that something I've noticed throughout this process is that neurodivergent people make friends and I think it's so seen in society that neurodiverse people can't make friends and it's like, nope, friendships can be actually a really good support network if a person finds them.* (Y‐RPer)

*So many interviews incorporate* a priori *themes often designed by and for non‐neurodivergent people, as a result, we may ask questions that may not be relevant to young people’s lives. The Y‐RPers shared knowledge with us, we implemented a simple “you said ‐ we heard” approach. Their views turned into novel interview items and tasks.* (Academic researcher).

There was a shared sense that leveraging the skills and experience of the Y‐RPers improved the quality of data, *leading to more nuanced and novel insights*. During the interviews, the Y‐RPers realised how sharing similar experiences with the interviewees helped them to emotionally identify with them. This empathic feeling helped the Y‐RP interviewers to further connect with the interviewees and created a relaxed, non‐judgemental atmosphere, in which interviewees felt comfortable to “open up a bit more and speak in confidence” and follow up with useful prompts to go deeper.

*I think you can tell during these interviews that when they say something and they don't necessarily open up massively, we can kind of understand it in a way that someone else might not. Or we can prompt them in ways that someone else might not.* (Y‐RPer)

Working together added an extra safety net to ensure that academic interviewers would not misunderstand or overlook important aspects of the interviewees' emotional experiences, as they had the Y‐RP interviewer to support them and follow up with relevant prompts to seek interviewees' perspectives. They also appreciated working with the Y‐RP interviewers and sought their recommendations to improve the interview experience for all sides.

*Both the Y‐RP co‐interviewer, and the young participant shared some common ground…There was some fundamental primary knowledge there, which the co‐interviewer had, and this gave them the benefit and the additional knowledge to go deeper into some questions and capture the nuances of the interviewee’s emotional experiences.* (Academic researcher)

Y‐RPers appreciated how their perspectives were “treated respectfully” and their contributions genuinely informed the analysis. Both Y‐RPers and ARs felt that involving neurodivergent young people in co‐analysis has the potential to lead to more “accurate” and “nuanced” representations of interviewees' emotional experiences. They commented on how co‐analysis challenged some preconceptions about the meaning of neurodivergent emotional expression they had previously entertained.

*[Co‐analysis] helps make sure the data is interpreted accurately. It is also significantly more ethical to extend the chance [to neurodivergent people] to participate in the research as co‐analysts instead of just subjects [of research].* (Y‐RPer)

*It helps reveal something new, adding nuance, or perhaps dispel myths around neurodivergence that are present in the academic literature.* (Academic researcher)

For additional quotes for each theme see the additional supporting information.

## DISCUSSION

In this paper, we describe the co‐development of a general qualitative research co‐production protocol based on RE‐STAR's participatory research framework (Sonuga‐Barke et al., 2024) and its application to the study of a specific topic—the emotional lives of neurodivergent adolescents. Post‐implementation feedback interviews and surveys with Y‐RPers, ARs and interviewees provided one of the first systematic investigations into the experience of these sorts of participatory processes from the perspectives of all participants (den Houting et al., 2021). This feedback highlighted several elements for successful co‐production.

First, is the importance of establishing certain *pre‐conditions* to ensure a successful participatory research experience. These included the need to establish trust and build rapport between ARs and Y‐RPers and for a flexible approach to working in a way that helps develop a sense of agency and builds autonomy. In line with previous research, building trusted, mutual relationships between academic and non‐academic collaborators is essential for ensuring respectful research into neurodivergence (Fletcher‐Watson et al., 2019; Pickard et al., 2022).

Second is the importance of *collaborating in neurodiversity‐inclusive ways*. Experience‐sensitive activities facilitated Y‐RPers’ input and improved the interviewee's experiences. Working alongside or being interviewed by someone who is neurodivergent can feel liberating. This is consistent with evidence that neurodivergent people feel more listened to and understood when interacting with other neurodivergent people or experience more positive feelings (e.g., enjoyment, comfort) during these interactions, which can in turn positively impact their self‐identity and wellbeing (Crompton et al., 2020; Milton, 2012; Pellicano et al., 2022).

Third is that all participants and researchers need to be supported to respond to the *new challenges* presented by co‐production and associated tasks and roles. Whether that was the Y‐RPers moving from advisors to researchers or the ARs becoming accustomed to new ways of working. Both groups recognised that implementing the protocol and fully integrating neurodivergent people required careful consideration of time allocation, human and financial resources and flexible working, even outside working hours. These constraints have often been reported in participatory research. They highlight systemic issues that should be addressed directly, to ensure participatory approaches are supportive within academic structures (Keating, 2021; Pickard et al., 2022).

Power hierarchies are inevitable even in participatory research of the sort discussed here. In RE‐STAR, power differentials in the AR‐YRP relationship have been systematically addressed by having weekly and monthly online reflexive meetings over 30 months where ARs and Y‐RPers discussed enablers and barriers for genuine involvement, openly and in goodwill. We also had open and, often, challenging discussions about optimal ways of conducting research involving people with different types of experiences, expertise and ways of working. By gradually building trust in each other, Y‐RPers began expressing a need for a deeper involvement throughout research, with the academic team offering consistent tailored support, training and guidance to ensure more authentic involvement (see Sonuga‐Barke et al., 2024 for a detailed description of the Y‐RPers’ journey from advisors to co‐researchers including intentionally addressing power issues with structural and relational practices that honour youth expertise). Navigating these central issues in supportive ways was essential to the Y‐RP's duty of care and the health of the study. Participatory research also requires high levels of adaptability to address the evolving needs and agendas of academic and non‐academic collaborators, particularly in transdisciplinary neurodiverse teams like RE‐STARs’. In RE‐STAR, establishing clear communication, creating safety in predictability and clear objectives for every meeting was a priority to ensure both comfort across neurotypes and scientific rigour (see also Fletcher‐Watson et al., in press).

Notwithstanding these challenges, all researchers felt that co‐production had the power to *improve translational research.* This was especially the case regarding the richness and nuanced nature of the accounts of neurodivergence and emotions produced. Y‐RPers were recognised as having a significant epistemic privilege. They bring valuable insider knowledge and expertise crucial to collecting richer and more genuine accounts from participants, who valued the neurodivergence‐sensitive interview activities and felt comfortable and heard when describing their emotional experiences in the presence of a neurodivergent co‐interviewer (Pickard et al., 2022).

Despite all its strengths, our study had some limitations. First, the sample size was small, but not unacceptable within the context of qualitative study and given the need for concerted and concentrated engagement to develop and implement the protocol. Second, the protocol's value was specifically established for studies with neurodivergent people aged 11–25 with a diagnosis of ADHD and/or autism, therefore it may require further adaptations when tested with other populations. Third, the evaluation was internally conducted by RE‐STAR members. It is therefore difficult to preclude the possibility that Y‐RP and ARs offered more positive responses as insiders in the project.

## CONCLUSION

Despite recent calls for a deeper involvement of neurodivergent young people across all research phases, from the co‐design of research to the co‐analysis of findings, few studies have described and evaluated a participatory framework that enables the gradual shift of young people from advisors to active co‐researchers. Importantly, such a methodological choice holds the promise of contributing to the development of alternative knowledge uptake models (Lyon et al., 2010).

RE‐STAR's participatory framework for qualitative research proved successful in involving young people more deeply and genuinely in research. This is currently being further tested in other RE‐STAR studies adopting different methodologies (e.g., interview‐, questionnaire‐, cohort‐, experiment‐, and neuroscience methods) and we hope this will be extended to other groups and settings to explore its generalisability and practical value for translational mental health research.

## AUTHOR CONTRIBUTIONS

**Myrofora Kakoulidou**: Data curation; formal analysis; investigation; methodology; resources; software; supervision; validation; visualization; writing ‐ original draft; writing ‐ review & editing. **Georgia Pavlopoulou**: Conceptualization; data curation; formal analysis; funding acquisition; investigation; methodology; resources; supervision; validation; visualization; writing ‐ original draft; writing ‐ review & editing. **Susie Chandler**: Data curation; formal analysis; investigation; methodology; project administration; resources; supervision; writing ‐ original draft; writing ‐ review & editing. **Steve Lukito**: Data curation; formal analysis; investigation; methodology; resources; software; supervision; validation; visualization; writing ‐ original draft; writing ‐ review & editing. **Maciej Matejko**: Data curation; formal analysis; investigation; methodology; resources; writing ‐ original draft; writing ‐ review & editing. **Isabel Jackson**: Data curation; investigation; methodology; resources; writing ‐ review & editing. **Beta Balwani**: Data curation; investigation; methodology; resources; writing ‐ review & editing. **Tiegan Boyens**: Data curation; formal analysis; investigation; methodology; resources; writing ‐ review & editing. **Sylvan Baker**: Methodology; writing ‐ review & editing. **Edmund J. S. Sonuga‐Barke**: Conceptualization; funding acquisition; investigation; methodology; supervision; visualization; writing ‐ original draft; writing ‐ review & editing.

## CONFLICT OF INTEREST STATEMENT

The authors of this paper have no conflicts of interest to disclose.

## ETHICAL CONSIDERATIONS

The study received ethical approval from King's College London's Research Ethics Committee (ref. HR/DP‐21/22–29361). Informed written parental consent and young person assent were secured for all participants before taking part.

## Supporting information

Supplementary Material

## Data Availability

Anonymised datasets will become available upon request where this is judged not to threaten the anonymity of participants.

## Appendix

The RE‐STAR team is: Edmund Sonuga‐Barke, Susie Chandler, Andrea Danese, Johnny Downs, Kirsty Griffiths, Myrofora Kakoulidou, Lauren Low, Steve Lukito, Angus Roberts, Emily Simonoff, Daniel Stahl, Anna Wyatt (King’s College London); Georgia Pavlopoulou (Anna Freud Centre and UCL) and Jane Hurry (University College London); Sylvan Baker (Royal Central School of Speech and Drama); Graham Moore, Amy Edwards (Cardiff University); Dennis Ougrin (Queen Mary University of London); Amanda Roestorf (Autistica), Niki Cooper, Julia Clements (Place2Be); Hannah Lathaen (ADHD Foundation) and Youth Researcher Panel members Jordan Altimimi, Beta Balwani, Saskia Barnes, Tiegan Boyens, Zoe Glen, Cj Harris, Charlotte Hillman, Luke Harvey‐Nguyen, Issy Jackson, Amber Johnson, Elisa Ly, Elizabeth Macauley, Maciej Matejko, Dorian Poulton, Anya Rose, Darren Webb, Archie Wilson.
