# Supplementary material for: Deepening the participation of neurodivergent youth in qualitative mental health research: Co‐development of a general approach and the evaluation of its implementation in a study on emotion
Source: JCPP Adv. 2024 Dec 7;4(4):e12287. doi: 10.1002/jcv2.12287 (PMC11669790; doi:10.1002/jcv2.12287)
Supplement: Supplementary file 1 — Supplementary Material [file JCV2-4-e12287-s001.docx]

**Supporting Information**

**Appendix S1.** Post-implementation questions on the experience of interview co-design, co-delivery and co-analysis.

**Appendix S2.** Example Y-RP materials that informed the co-design of a new interview schedule on emotional responses in ADHD and autism.

**Appendix S3.** Co-developed interview schedule.

**Appendix S4.** Themes and additional quotes for Y-RP researchers, academic researchers and interviewees.

**Figure S1.** A poem created by a Y-RPer to explain how they get ‘unstuck’ from difficult emotions.

**Figure S2**. A colourful shirt that shows how by brain works … bright … unconventional.

**Figure S3.** My ADHD and dyslexic mind.

**Table S1.** Themes and additional quotes on the interview co-design, co-delivery and co-analysis experiences.

**Appendix S1.** Post-implementation questions on the experience of interview co-design, co-delivery and co-analysis.

**Post-implementation questions on interview co-design experience**

For academic and Y-RP researchers

- How did you find the interview co-production process?

For Y-RP researchers only

- How was the process of meeting with academic researchers and co-defining the topic of emotional regulation?
- How was your experience of co-producing the interview schedule (e.g., interview questions, video vignettes)?
- How was it for you to discuss pre-study considerations with academic researchers?
- What did you enjoy more or less about the interview co-production process? Did you feel as a co-researcher from the beginning? Why/why not?

**Post-implementation questions on interview co-delivery experience**

For young people and all interviewers (academic researchers + Y-RP)

- What did you like about the interview process?
- What didn’t you like about the interview process?
- Did having a co-interviewer with their own neurodiversity made a difference to the interview experience for you? In what way?
  - 3a. do you think being a neurodivergent interviewer makes a difference to the interview experience for you? In what way?
- What do you think this (having members of the neurodiverse community interview neurodiverse young people) adds to the interview?
  - 4a. what do you think being neurodivergent, interviewing neurodiverse young people adds to the interview?
- Do you think this makes the interview process more challenging in any way? How?

For Y-RP interviewers only

- What do you feel you can take away from this experience … (e.g., the training and experience in terms of adding to your CV, opportunities for future work)
- How do you think the training and prep. could have been improved? (For co-interviewers). What did you like about the training/prep?

**Post-implementation questions on the interview co-analysis experience**

For academic and Y-RP researchers

- How did you find the analysis workshops/training?
- Did you learn anything from co-analysing the interviews e.g., during meetings to discuss coding?
- How was your experience of planning and meeting in pairs and groups?
- In what ways do you feel that you contributed to the analysis? Please share some examples below.
- How has the experience of co-analysing the interviews with others (e.g., neurotypical, neurodivergent) impacted your thinking and coding?
- What impact do you think you had on the analysis and on the others?
- How did the experience of co-analysis impact you on a personal level? For example, how was it for you to analyse the interview of another neurodivergent young person about their emotions?
- Is co-analysis important? Why or why not?
- What can co-analysing interviews with neurodivergent young people offer to the academic analysis?
- We ran two different types of co-analysis. Do you prefer whole group meetings looking at codes and clustering codes/nuancing meanings/suggesting themes or working from scratch on transcripts and then in pairs to co-analyse?
- What suggestions do you have for academic researchers who want to involve neurodivergent young people during data analysis?

**
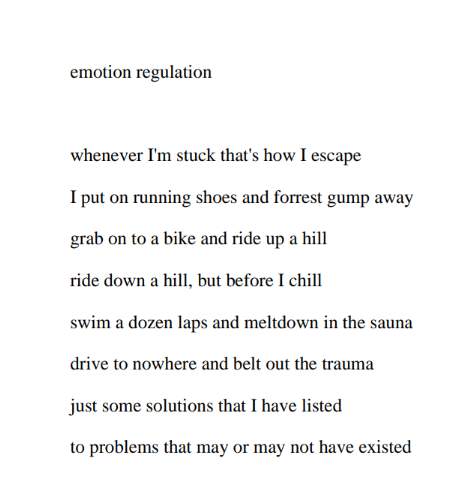
** **Appendix S2.** Example Y-RP materials that informed the co-design of a new interview schedule on emotional responses in ADHD and autism.

FigS1 A poem created by a Y-RPer to explain how they get ‘unstuck’ from difficult emotions (Y-RPer with a diagnosis of autism, age 24)


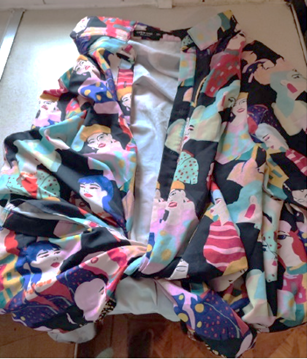

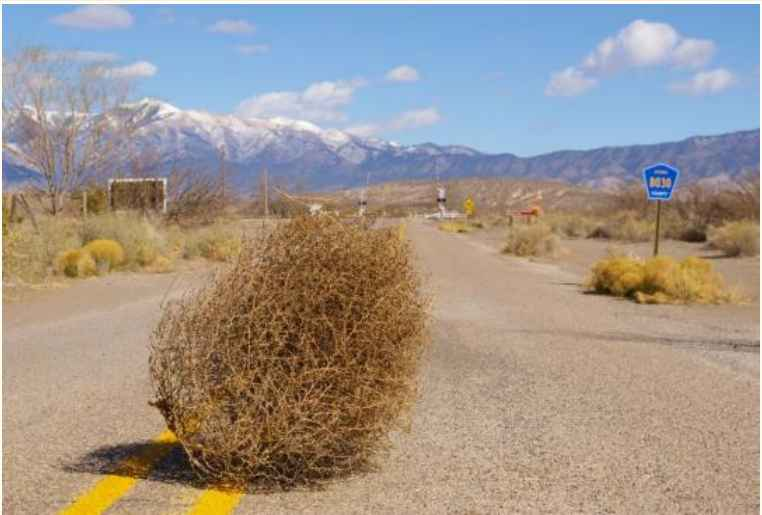


FigS2 A colourful shirt that shows how by brain works … bright … unconventional. Loud shirt which is fastened with a knot because the buttons aren't there (i.e. my brain) (Y-RPer with a diagnosis of ADHD and autism, age 24)

FigS3 My ADHD and dyslexic mind. Many channels of thought, all interweaving. The tumbleweed/me can be blown about in different directions all the time, and at different paces , but the weed manages roll along through the road/life nevertheless. (Y-RPer with a diagnosis of ADHD, age 22).

**Appendix S3.** Co-developed interview schedule.

# MY EMOTIONS AND ME

**PROPOSED INTERVIEW SCHEDULE**

*Thank you so much for taking part in this interview. Today, we will go through different topics, and look at anything you have created for today’s session. In particular, we would like to have a chat about your experience of strong emotions and how you manage them in different situations.*

*We will not share what we talk about today with your parents or anyone else unless we are concerned about your safety.*

*If there’s anything I ask that you don’t want to answer, that is absolutely fine. Please let me know and I’ll move on to the next question. Also, if you want to stop completely at any point that is okay. Just let me know. Does that all make sense?*

*I will start with a few questions about yourself.*

1. **ICE-BREAKERS**
2. **Can you tell me how old you are?**
3. **What are your hobbies/interests?**
4. **Can you describe what a typical day is like for you?** *[prompts for morning/school/after school/evening].*
5. **MY EMOTIONS AND ME: CREATIVE TASK**

**Description:** In this task, the participant will present an art/craftwork that we have assigned for them to do at home before the session. We will discuss with the young people how this art/craftwork represents their emotions.

**Instruction:** *Now, we’ve asked you to make something to express the times that are calming, reassuring or difficult and upsetting or both, and reflect on your reactions. Shall we look at what you’ve made?*

1. ***What is it?***
   1. *Please read/show/describe to me what you’ve written/made.*
   2. *Does it have a name/title (e.g., X)?*
2. ***How does this (or X) relate to you being feeling this emotion?***
   1. *What are the situations that X remind you of…?*
   2. *What kind of emotion do you feel then?*
   3. *What makes it emotional for you? How is it different from the usual emotion you have?*
3. ***How long do you feel that way?***
   1. *Did it keep going? What kept it going?*
   2. *Did the feeling change over time?*
4. ***How do these feelings stop?***
   1. *Do they go away on their own?*
   2. *Do you have to do anything to handle them? What are they?*
5. ***What else can you tell me about X?***
6. **MY EMOTIONS AND ME: RESPONDING TO SCENARIOS**

**Description:** Participants will be asked to select a vignette from a selection (of up to 8) presented on their screen. These will be displayed as coloured cards on the screen with a short title. Potential vignette topics are listed below. When the young person clicks on a card, a short video clip will play (for 20-30 secs). In the clip, a young person with a diagnosis of ADHD and/or autism will appear and briefly narrate a scenario. This will include captions. At the end of the video, a photo or drawing will appear inviting the participant to consider whether this situation is relevant to their own lives, and how this (or a similar situation) would affect them. We will stay curious about what it is like for the young person, and explore what the internal and external factors are in each situation.

We will then go through the questions below with the young person.

**Topics of vignette:**

| - sensory discomfort situation - being expected to understand/follow social rules or read other people’s minds - frustration tolerance situation - invalidation situation/dismissal from others - responding to demands/showing compliance - limited options to make choices - an unexpected change in routine/uncertainty - masking or feeling of not belonging - engaging or lack of engagement with focused interests - misunderstanding social situations - paying attention to details that others don’t | - not paying attention to details that others do - taking turns in a conversation or game - paying attention for a long time to the least interesting subject - meeting deadlines - acting fast- then regretting - thinking of what others might be thinking of me in a social situation - negotiating with parents - negotiating with teachers - video gaming situations - interaction with peers at school |
| --- | --- |

**Instruction**: *Ok, I will now ask you to look at different scenarios and think: whether you have ever been in similar situations; what your emotional reactions were; and how you managed (or didn’t) to stop these emotions. Let’s start with you choosing which scenario we would like us to start with by clicking on the box of your preference. Once you click on the box, a short clip will play presenting you with a scenario.* *I will ask you 5 questions for each card. Ready? (showing card/slide/vignette). There are no right or wrong answers.*

**Example video vignette for the school context**


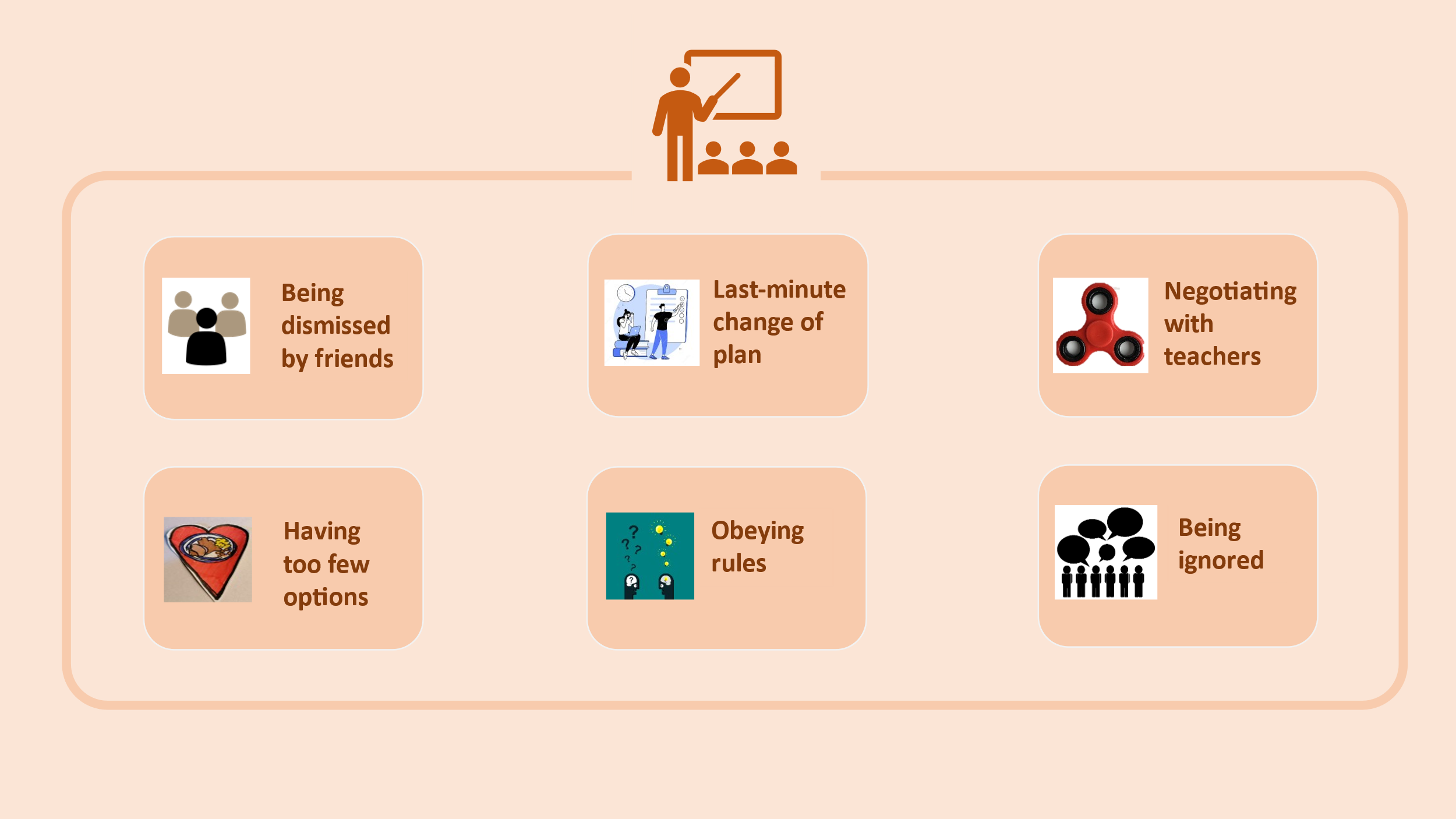


(last-minute change of plan, vignette narrated by a Y-RP member)

*At my school, students go to registration every Friday morning and then to Maths. I’ve got all my books ready and prepared for. But today, we are told last-minute that there is an assembly we have to attend. So now, everything that I’ve planned has got to be changed.*

**Example video vignette for the home context**


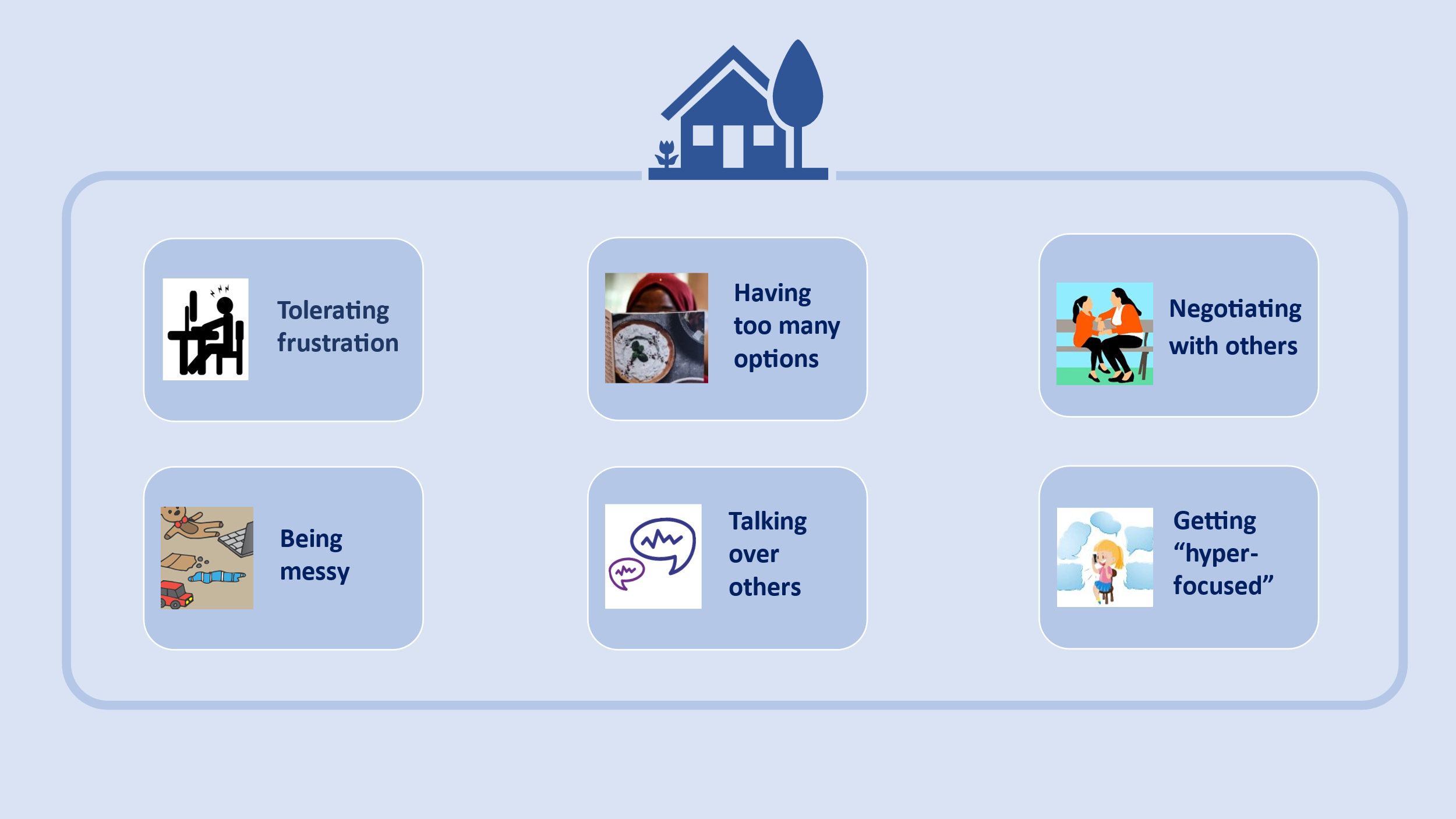


(being messy, vignette narrated by a Y-RP member)

*I find it hard to keep my room tidy. Things often go missing and I will be searching for them for hours. The worst is if something goes missing before I need to get somewhere. I’ll be running from one room to another and there’ll be a lot of shouting.*

**Example video vignette for different contexts**


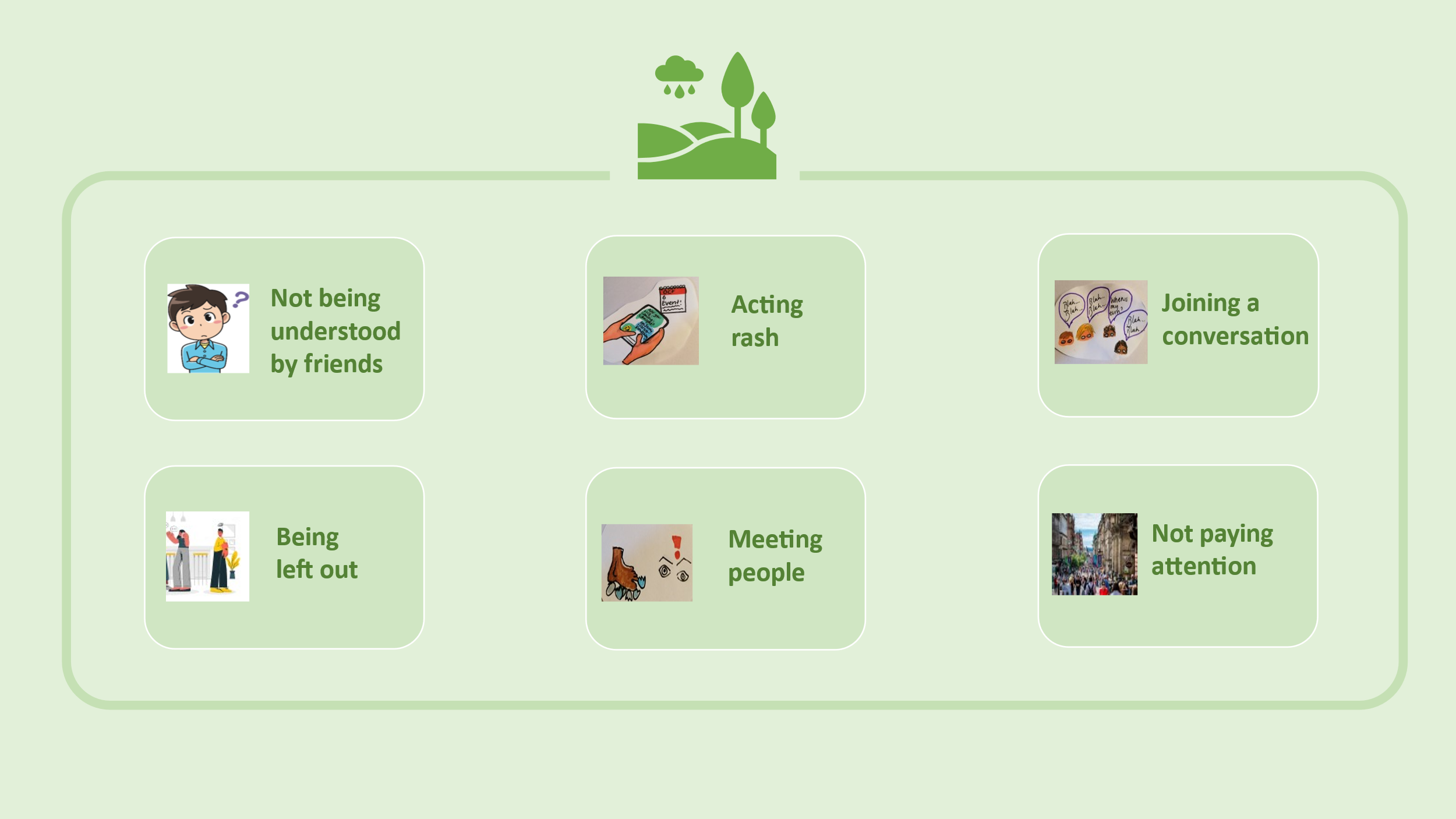


(meeting people, vignette narrated by a Y-RP member)

*Meeting people, especially new ones, is tricky for me. Sometimes I feel like I am “walking on eggshells” to avoid saying the wrong thing or offending someone without realising it.*

1. ***Is that situation familiar to you?***
   1. Have you ever been in a situation where …
2. ***What's your emotional reaction in a similar situation?***
   1. *How do you feel then? You can use the emotion chart to help describe your feeling.*
3. ***How do you express your emotion in that situation?***
4. ***What is your reaction to other people when you feel that way?***
5. ***What is the reaction of others to you when you feel that way?***
6. ***Have you ever managed not to feel that way?***
   1. *Could you change your feelings once they have started? What had helped you to stop the feelings before they started?*
   2. *Did your environment allow you or stop you to do what you needed to stop these feelings?*
7. ***How was it after you had experienced the feelings?***
   1. *For example, some young people have told us they couldn’t get to sleep that night thinking about this. What about you? What do you do after a similar situation? What would you do?*
8. **MY EMOTIONS AND ME: THINKING ABOUT DIFFERENT EMOTIONS**

**Description:** In this segment, we will show a colour-coded chart containing words (and/or emojis) that describe different clusters of emotions (see below). We will ask the young person to pick one emotion from each colour and go through the questions below. If appropriate, we could consider a participatory approach, e.g., by presenting the task in a playful manner, to help the young person access the interview. For instance, we can ask the young people to decorate the emoji or to match the emoji with a colour zone/emotion. We will make no prior assumption of what the chosen emotion means. Rather, we will stay open to the young person’s definition. We will stay curious as to what it is like for the young person and explore what the internal and external factors are, i.e., check if the person is able to recognise traits in themselves and others/environment.

**GREEN: Calm, Happy, Focused, Ready to Learn/ Play/Interact with others, Content, Excited**

**BLUE: Sad, Bored, Tired, Sick, Disappointed**

**YELLOW: Worried, Silly, Frustrated, Stressed, Hurt, In pain**

**RED: Mad, Upset/Loud, Scared, Angry, Out of Control, Irritable**


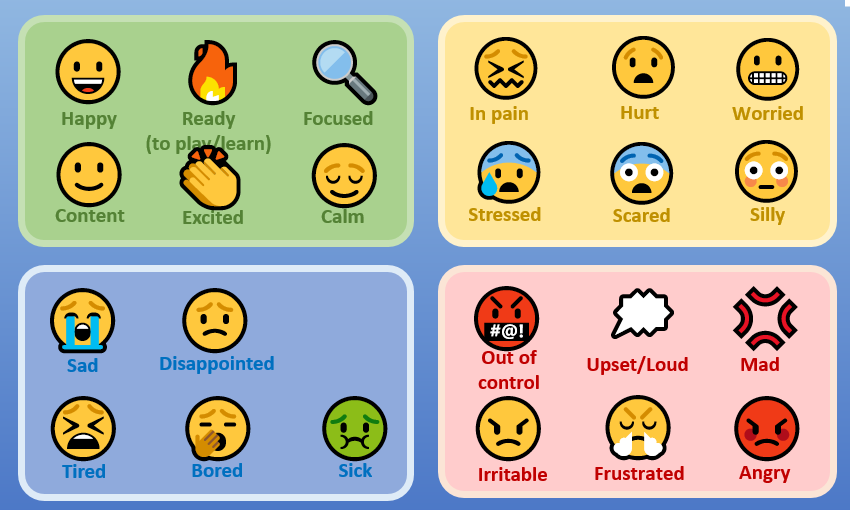


**Instruction:** *I will show you some words that express different emotions. I will ask you what situations bring these feelings at home, school or elsewhere, how long these feelings last, how you handle them and how you stop them. Let’s pick a colour.*

1. ***So, what was it in your life at home or school that has made you feel X?***
   1. *Or can you tell me about a time at home or school when you felt X?*
2. ***Are there times/occasions that you are more likely to feel X? Is it at home or school? Is it during or after an event?***
3. ***What did you exactly feel at that moment? Where in your body did you feel it/how did your body react?***
   1. *Did you feel anything in your body when you felt X?*
4. ***How long did the emotion or feeling last?***
5. ***How did you handle this emotion? What did you do?***
   1. *What did you do when you felt like this?*
   2. *How was this related to how you experienced X?*
   3. *How did you respond to other people while you were feeling this emotion?*
   4. *How did others respond to you?*

*Let’s pick another emoji* (Remember to repeat these questions for every colour zone).

**Useful additional general prompts (see the above for examples of when to ask the prompts):**

- *How is this related to how you experience X?*
- *How does that make you feel?*
- *What do you do when you feel like this?*
- *Can you tell me about a time when…?*
- *Reflect by repeating a word and wait to hear clarification.*
- *Can you go back and tell me a little bit more about a time when…*
- *What do you mean by “bad”? Help me understand what “bad” looks like for you.*

1. **MY EMOTIONS AND ME: RECONSTRUCTING QUESTIONS AND WHAT HELPS**

**Instruction:** *Now, let’s think about things that can bring emotions such as too much excitement or too much anger back to a level so we can think clearly and manage the situation.*

1. ***What are the things that boost our mood?***
2. ***What gives you a sense of achievement?***
3. ***What things around you can help you manage too much excitement or too much anger?***
4. ***Have you ever been in a situation where the way you chose to manage your emotions was not accepted by others? Why do you think this happened?***
5. ***What are the things you wish your teachers, parents or friends understood better about how you manage your emotions such as over-excitement or anger?***
6. ***What could they do to help?***

***Is there anything that I haven’t asked you about X that you’d like to tell me?***

**(Discuss reimbursement with the YP – thank you- remind support contact lines)**

**Appendix S4.** Themes and additional quotes for Y-RP researchers, academic researchers and interviewees.

**Table S1. Themes and additional quotes on the interview co-design, co-delivery and co-analysis experiences**

| **Themes** | **Example quotes** |
| --- | --- |
| **Theme 1: Pre-conditions for genuine collaboration between academic and Y-RP researchers**  Sub-theme 1: Trust, mutuality & attunement  Sub-theme 2: Autonomy & flexibility  **Theme 2: Working together in neurodiversity-inclusive ways**  Sub-theme 1: Engaging through experience-sensitive activities  Sub-theme 2: Learning from others  Sub-theme 3: A sense of comfort & validation  **Theme 3: Taking on new challenges & managing struggles**  Sub-theme 1: Shifting power dynamics  Sub-theme 2: Embracing diverse ways of thinking & working  Sub-theme 3: Managing duty of care, planning time & expectations  **Theme 4: Improving translational research**  Sub-theme 1: Developing new research hypotheses  Sub-theme 2: Collecting nuanced & novel data | “Initially there was a bit of anxiety of not knowing if I’m doing this right and sharing it with others, but I eased into it with time and feedback.” (Y-RPer)  “The academic researchers were very responsive to criticism of research in the field: mainly how often the voices of the people it claims to try to understand are excluded, and how our experiences are explored primarily through the questions neurotypical people have about what they perceive as "deficits".”  (Y-RPer)  “Not rushing the process and having time to know the Y-RP over a period of many months was key in creating an equitable partnership. Relationships and trust can be forgotten in big busy projects, trust was key to unlock the potential of the young people.” (AR)  “I would imagine that having a neurodivergent co interviewer would help the young person feel more comfortable. For example, when [the Y-RP interviewer] decided to disclose their diagnosis in the beginning of the interview, I could see the young person smiling. I think this put them more at ease that they were in a safe environment.” (AR)  “I do also like the fact that we kind of met before and we decided on how to split stuff so there was clear structure to it that you know you could rely on if you didn't quite know what to say.” (Y-RPer)  “…We do have a clear framework of tasks, and obviously our example questions, but we're also not expected to just stick to those. Like we are allowed to sort of follow things if were curious about something we think is relevant and kind of bring our own experience to that. If we think it matters and the interviewers kind of let us do that.” (Y-RPer)  “I felt we were able to contribute changes to make sure the schedule was directly answering the research question whilst making sure it was presented/asked in a way that best suited the young people and in turn (hopefully) led to more relevant responses.” (Y-RPer)  I think at least in the beginning, Padlet worked better than speaking for us. Speaking in bigger groups, especially when you don’t know others, can be stressful, since it’s like directing everyone’s attention on yourself (it took me some time to ease into it in our meetings). But on Padlet everyone takes their time focusing on what they want to express, it’s pretty much anonymous, so it takes away the stress of speaking. Then, if someone wants to say more on the mic, they’re free to do it and they have the Padlet note to base from. I remember the notes popping up rapidly in Padlet and it felt good to be in a group that freely expresses their neurodivergent experiences. (Y-RPer)  “It wasn't solely about evolving appropriate interview questions, it was about a deeper consideration about how it might feel to be asked questions, and what might happen after the interview had taken place. It was in a sense, a more holistic consideration of more than the dataset, which is clearly very important, that encompassed empathy and ascetic of care for the participants.” (AR)  “We listened carefully to the experiences of Y-RPers during the creative activities and then we summarised key themes they said - we heard. Then we brainstormed ideas on how the concepts they coined can become part of the interview schedule. (AR).  “I liked the activities. The photo ones, and the one’s where I'd watch the video, and I would explain how I related to it. Because I think it just feels nice to know other people who have been through similar experiences.” (Interviewee)  “[I liked the little videos and the pictures of emojis] If I have some kind of thing to start talking about, like a basis something there to help me start talking, it can help.” (Interviewee)  “It's interesting to me to get to know neurodivergent young people in this way. And I think we've learned from others, they have a range of different go to things to cope with, being overwhelmed or with difficult emotions.” (Y-RPer)  “I think it's a nice experience to have this experience of talking about difficult things with neurodivergent young people. I think it's sort of makes me, I don't know, maybe it makes me a better listener or helps me be more confident. Talking about difficult things.” (Y-RPer)  I can't remember if they had autism or ADHD, but either way, they weren't very stereotypical, I wouldn't say. So sometimes actually some of the things that people presumed they would struggle with they wouldn’t. It is important to hear the other sides, (you hear people say that those with ADHD or Autism will struggle with something you’d expect), but actually talking to them, you realise actually there's other struggles or things that are not so widely considered as a normal thing to struggle with (Y-RPer)  “The questions were good overall. But it's good because they kinda understand a bit more, which leads to further questions and more interesting stuff to talk about, I guess.” (Interviewee)  “It makes it easier to chat about stuff if you know someone the same diagnosis.” (Interviewee)  “It's just because we had some kinda relatable experiences. So it helped that (the co-interviewer) was able to sympathise. So he could understand. Like he's had similar experiences and also like with general things with like not being understood quite a lot of the time. We can kinda relate on that.” (Interviewee)  “At the beginning of the co-production, I felt like a subject rather than a researcher... As the project progressed and more information was shared with us and the Y-RP was offered more opportunities to get involved and provide input to the project the more I felt like a co-researcher.” (Y-RPer)  I’m not sure if I felt as a co-researcher right away, as the first meetings were to a large part about getting to know what RE-STAR is, what the idea for our involvement was, meeting the new people etc. Also I guess at the very beginning we were sort of like participants in a pilot study. When we were giving feedback about the schedule, it was still more of an advisory role. I’m thinking that co-interviewing people was a big milestone for me in this regard. I felt that we are actually getting into the role of researchers, that you gave us a lot of trust to do this and that we’re doing something unique. (Y-RPer)  I definitely didn’t feel like a co- researcher from the beginning for me it felt more an advisor, or someone coming to observer the process and give any feedback. It took time to feel like a co-researcher and even learn what that means. It felt like stepping into a new world and going on an adventure. Finally, I think the most enjoyable part for me was seeing of the development of not only the interview schedule but also us as a group. There was massive evolution throughout, the interview schedule developed into something really considerate and my understanding of the project really changed. (Y-RPer)  “Power dynamics are inherent in participatory research even if academic researchers have the best intentions. It was really important to ensure the gradual shift of Y-RPers from advisors to co-researchers. The Y-RPers showed a great enthusiasm for a deeper involvement in research. We wanted to address this sincere request for meaningful involvement. For example, we had monthly meetings with them to discuss ways of involvement and designed different training to support them in taking the co-researcher role. They also started attending key meetings with co-investigators and stakeholders (AR)  “It was important to attune with young people’s aims and objectives for any kind of meaningful action to take place. I would say listening deeply and then giving them an organised space to take action is what worked very well. Discreet and individualised support was always available… also apologising and being accountable for any confusion or inconsistency in the structure was important. (AR)    “I think my coding was significantly improved for doing it with others, especially those with different neurotypes to my own.” (Y-RPer)  I felt I really quickly learnt not only the analysis process but also the thought process to use in it. I was actually learning research methods at that time at university and had no understanding of the method when taught at university if anything was left more confused. However in less time I just got it and realised how easy it is once you know the process. (Y-RPer)  “[The whole process] might take some time; the organization, scheduling of appointments might take some extra time but, yeah, it's a good thing to learn; it's a new learning experience for all of us to try to be more flexible.” (AR)  “I think it was quite a difficult interview to carry out and I was very lucky that [Y-RP interviewer] was very experienced in that regard. And, you know it could have been completely different.” (AR)  “Having a Y-RP interviewer helped to refresh the interview process. The way that [the Y-RP interviewer] asked the questions…So, if something didn't work with the way I asked the questions I would always have them to back up on me.” (AR)  “Young people can bring a diverse set of experiences and skills which can play a central role during analysis. Academic researchers should be ready to be challenged - there isn't only one way of interpreting things and reaching consensus shouldn't be the goal.” (AR)  “The transcripts themselves were quite interesting to me, but the coding itself was tedious at times, especially when having to go back to something many times, over a longer period.” (Y-RPer)  “To set up this interview was so difficult to begin with, because our timetabling between the [Y-RP interviewer] and myself do not overlap that much and so it was more logistic difficulties rather than anything.” (Y-RPer)  “I think it's taken a long time. But I'm very aware that we've been learning the process anyway, like we all went through training… So I think we've already been on a lot of learning journey.” (Y-RPer)  “I'm guessing maybe just more opportunity to practise it with others [would be helpful]. Because when we just started practising the schedule between us, between the Y-RPers, it already helped a lot. And then when I started to when I started meetings with X [academic interviewer] and the practice, I think it was all really, really good.” (Y-RPer)  “I think a solid structure with clear aims/instructions and focus on answering research questions are very essential for qualitative research work. I am still discovering that.” (AR)  The co-analysis process can be time-consuming and requires human power so that researchers can share workload and meet deadlines as expected. Involving young people in co-analysis may also require various rounds of negotiations with ethics committees.” (AR)  “The young person started telling story…and then they started crying and that was a challenge… I felt immediately worried about everyone's well-being and that would be on me, you know, as a person who's leading the interview, in a way. I didn't want to expose [the Y-RP interviewer] in their first interview with that kind of difficulties, and yeah so I think that's something that I felt uncomfortable about.” (AR)  It was refreshing to chat with other neurodiverse people about emotional regulation to realise we had shared experiences. It helped to see that emotional regulation was limited/impacted by our environments, so removed blame on us as individuals. It was also nice to be discussing this for a purpose which can go forward and hopefully support others with their emotional regulation (Y-RPer)  “We're showing our perspective to the world almost. And actually a neurotypical person could alter that [perspective]. I think that's what we're challenging as a whole research project, I would say. It’s actually the idea of what perspective are we looking at all these things from? And in the past it's very much been a parent (which is) a neurotypical view.” (Y-RPer)  [The Y-RP interviewer] themselves was such a super interviewer. I liked their way and sometimes they would get into a particular question and then try to keep an open mind about things and ask a follow up question that maybe I haven't necessarily thought about.” (AR)  “[Working with neurodivergent researchers] ensures the analysis is correct, that themes are…correctly interpreted and that the data collected is actually relevant and helpful to the community.” (Y-RPer)  “It is understood differently by somebody who is neurodivergent themselves, even if it's not the same type of neurodivergence... versus when you speak to a neurotypical person like they can have all the best intentions in the world, record it faithfully and try to draw conclusions possible from it. But you can still kind of sense and see that they don't quite understand it necessarily. And when you're being interviewed for research, I think knowing that there is someone who actually does get it, even if their personal experiences different from yours. Importantly, it does matter to people.” (Y-RPer)  “I think actually if I wasn't neurodiverse, I might be going for a different set of detail, but because of our understanding or I can see more where they're coming from, even if I have no knowledge or experience like that, I sort of semi get where they're coming from more. So I think it's sometimes aided the questions and my understanding of what they're saying.” (Y-RPer)  “I felt more relaxed having a person with lived experience in the room. I felt more confident that we are going to get the young person's story right, that we're not going to misunderstood them, that I have another co-interviewer in the room asking further questions to unpack things.” (AR)  “It was fascinating to be able to check the usefulness and accuracy of the language we use to describe themes, how to declutter themes and how to express them in ways that truly serve the community. The conversations were crucial to move from descriptive to more latent codes.” (AR) |

Y-RP = Youth Researcher Panel (also called “Y-RPer”); AR = Academic researcher
